# Agreement and reproducibility of radiological signs in NEC using The Duke Abdominal Assessment Scale (DAAS)

**DOI:** 10.1007/s00383-016-4022-y

**Published:** 2016-11-14

**Authors:** Karolina Markiet, Anna Szymanska-Dubowik, Iwona Janczewska, Iwona Domazalska-Popadiuk, Anna Zawadzka-Kepczynska, Agnieszka Bianek-Bodzak

**Affiliations:** 1 II Department of Radiology, Medical University of Gdansk, Gdansk, Poland; 2 Department of Neonatology, Medical University of Gdansk, Gdansk, Poland; 3 Department of Radiology, Medical University of Gdansk, Smoluchowskiego 17, 80-214 Gdansk, Poland

**Keywords:** Abdominal X-ray, NEC, Duke Abdominal Assessment Scale, Intraobserver agreement, Interobserver agreement

## Abstract

**Purpose:**

Necrotizing enterocolitis (NEC) is associated with high morbidity and mortality. Abdominal radiography is currently an imaging modality of choice in NEC. Recently, a numeric scale of radiological signs in NEC—The Duke Abdominal Assessment (DAAS) was introduced. The aim of this study was to measure the intra- and inter-observer agreement on the radiological signs of NEC according to DAAS to access the feasibility of this scale.

**Materials and methods:**

We have retrospectively analyzed 87 radiographs performed in a group of 43 high-risk neonates with suspected NEC. Radiographs were assessed by 6 independent observers: two pediatric radiologists, two radiology residents, and two neonatologists. Data were analyzed using *κ* statistics as a measure of intra- and inter-observer agreement.

**Results:**

Fair-to-good intra-observer agreement was noted for all but one of observers. However, with the wide range in *κ* values, we found only fair inter-observer agreement detecting signs of NEC according to DAAS. There was a higher intra-group agreement in radiology practitioners, with the highest among experienced pediatric radiologists.

**Conclusion:**

However, with high observer variability in interpretation of all radiologic signs, we did not confirm that Duke Abdominal Assessment Scale could reliable facilitate reporting of abdominal radiographic findings in neonates with suspected NEC.

**Electronic supplementary material:**

The online version of this article (doi:10.1007/s00383-016-4022-y) contains supplementary material, which is available to authorized users.

## Introduction

Necrotizing enterocolitis (NEC) is a severe inflammatory process of the gastrointestinal tract in neonates and infants and one of the most common abdominal emergencies in this age group, especially in premature neonates. It is associated with high morbidity and mortality (20–45%), higher in neonates with very low birth weight and those presenting with perforation [1–5]. Therefore, the early and correct diagnosis is of utmost importance. In addition to the clinical symptoms and laboratory tests results, abdominal sonography (US) and plain abdominal radiography are used to diagnose NEC in clinical practice [3–5].

The value of ultrasound in the diagnostics and follow-up of NEC is discussed more and more often. The sensitivity of free air at abdominal radiography as a positive sign for severe NEC was 40% compared with the 100% sensitivity of the absence of flow at color Doppler US [6, 7]. Recent studies by Muchantef et al. and Dilli et al. comparing sonographic and radiographic imaging features in NEC confirmed the above-mentioned findings and proved that US is superior to abdominal radiography in evaluating focal fluid collections and that it shows greater sensitivity for demonstration of free peritoneal gas [8, 9].

Despite indisputable advantages of abdominal sonography, abdominal radiography is currently an imaging modality of choice in evaluation and follow-up of neonates suspected of or diagnosed with NEC [1, 5, 10]. The presence of focal and diffuse gaseous intestinal distention, air–fluid levels, bowel wall thickening, ascites, pneumatosis, portal venous gas, and pneumoperitoneum is assessed most often [10–12]. Recently, a ten-point numeric scale of radiological signs in NEC has been introduced into clinical practice—The Duke Abdominal Assessment Scale, in an attempt to standardize the terminology in reporting abdominal radiographic findings in NEC and to facilitate communication between radiologists and referring neonatologists [13]. Authors found significant intra-observer and inter-observer agreement between study participants [13].

In a frequent daily situation of no possibility to have a consultation with a pediatric radiologist, an introduction of a reporting system that simplifies and organizes radiological signs of NEC seems necessary and reasonable for many clinicians, especially in a situation when an immediate consultation with a pediatric radiologist is impossible.

The aim of this study was to measure the degree of radiologists’ and neonatologists’ intra- and inter-observer agreement on the radiological signs of NEC according to The Duke Abdominal Assessment Scale to access the feasibility of this scale in daily practice.

## Materials and methods

The study was conducted as an analysis of plain X-rays performed in a group of 43 high-risk neonates (21 males and 22 females) with suspected NEC, admitted to The Neonatal Intensive Care Unit (NICU) of The University Hospital in years 2005–2009. Forty-two infants were born prematurely (25–34 Hbd), one was full term (42 Hbd), all presented with a low birth weight (480–2000 g, average 1123.7 g), and 28 were delivered by cesarean section. The initial clinical diagnoses of the newborns are presented in Table 1. According to modified Bell’s staging for NEC, 12 newborns were initially defined as definite and advanced necrotizing enterocolitis [4, 14]. In this group, 3 deaths were reported at NICU, 9 patients were transferred to Pediatric Surgery Ward, of whom 5 presented with clinical symptoms of perforation; 8 underwent surgery. Twenty-two infants were discharged home and two were transferred to Pediatric Care Ward due to congenital defects and TORCH infection. The bioethical committee granted a waiver of informed consent due to the retrospective design of the study.

**Table 1:** Initial clinical diagnoses in 47 neonates and infants with suspected NEC

Diagnosis	Number of neonates
Respiratory distress syndrome	36
Pneumonia	8
Sepsis (verified by positive blood cultures)	8
Patent ductus arteriosus	3
Persistent pulmonary hypertension	1
Atrial septal defect type 2	2
Bradycardia	3

Eighty-seven radiographs were selected for the analysis. All examinations were performed in single anteroposterior projection in an upright position. X-rays were anonymized prior to evaluation and subsequently assessed by six independent observers blinded to clinical data in two sessions with time interval of 4 weeks; observers did not have access to the results of their previous interpretation during the second assessment. Besides two principal investigators, both experienced pediatric radiologists (O5, O6), two radiology residents in their first three years of training (O1, O2), and two board certified neonatologists (O3, O4) were recruited. All participants underwent proper, 3-month training in evaluating abdominal radiographs in accordance with DASS reporting system.

The selected X-rays were evaluated in the same room by all observers, under comparable illuminating conditions. Radiographs were assessed according to a ten-point numeric scale of radiological signs in NEC—The Duke Abdominal Assessment Scale (DAAS)—Table 2 [13].

**Table 2:** Abnormal radiographic findings in neonates and infants with suspected NEC—Duke Abdominal Assessment Scale (DAAS); reprinted from [12]

Score	Findings
0	Normal gas pattern
1	Mild diffuse distention
2	Moderate distention or normal with bubbly lucencies likely corresponding to stool
3	Focal moderate distention
4	Separation or focal thickening of bowel loops
5	Featureless or multiple separated bowel loops
6	Possible pneumatosis with other abnormal findings
7	Fixed or persistent dilatation of bowel loops
8	Highly probable or definite pneumatosis
9	Portal venous gas
10	Pneumoperitoneum

Table reprinted from [12]

Statistical analysis was performed with the use of commercially available software (http://www.r-project.org/). Data were analyzed using the *κ* statistics as a measure of intra- and inter-observer reliability, as well as intra-group reliability. Intra-observer agreement was determined from the scoring system by comparing data obtained from the same observer at two reading sessions. Inter-observer reliability was evaluated by means of comparison of data from pairs of observers for each of the observers’, respectively, at either session. Intra-group reliability was assessed by means of comparison of the results from the first reading sessions for each pair of observers: pediatric radiologists, radiology residents, and neonatologists. *κ* values and *κ*-weighted values were calculated according to Cohen. Kappa’s values range from −1 to +1. −1 stands for maximal disagreement and 0 means that observed agreement equals chance agreement, while +1 corresponds to maximal agreement beyond chance. Kappa (*κ*) values can also be interpreted as a percentage of agreement between observers (*κ* value of 0.38 equals 38% agreement between observers). The level of agreement was measured according to Altman: *κ* < 0.20 (poor agreement), 0.21 < *κ* < 0.40 (fair agreement), 0.41 < *κ* < 0.60 (moderate agreement), 0.61 < *κ* < 0.80 (good/substantial agreement), and *κ* > 0.81 (excellent agreement) [15, 16]. Kappa weighted (*κ*_w_) includes the degree of disagreement in the calculation and a value >0.5 corresponds to an acceptable degree of agreement [17].

## Results

In all 12 neonates except for one, diagnosed as either definite or advanced NEC, the final scores were 5 or more. The radiogram of this neonate with NEC I° according to Bell’s staging was acknowledged as normal (score 0). In most cases with NEC, the readers acknowledged the radiograms six points that meant probable pneumatosis.

*Intra-observer reliability* Table 3 presents mean *κ* and *κ*-weighted values for intra-observer agreement. Fair-to-good agreement was noted for all of the observers apart from one of the radiology residents (O2).

**Table 3:** Intra-observer reliability

Observers^a^	*κ*	*κ* weighted
O1	0.7198	0.8140
O2	0.1222	0.1830
O3	0.3282	0.4717
O4	0.3458	0.5233
O5	0.4683	0.5543
O6	0.6240	0.8050

^a^ *O1 and O2* radiology residents, *O3 and O4* neonatologists, *O5 and O6* pediatric radiologists

*Inter-observer reliability* With the mean *κ* and mean *κ* weighted values for inter-observer agreement varying widely between 0.0323–0.2920 and 0.0367–0.5353. Accordingly, there is only fair agreement.

*Intra-group reliability* Mean, minimum, and maximum *κ* and *κ*-weighted values are shown in Table 4. There was a higher intra-group agreement in radiology practitioners, with the highest one among experienced pediatric radiologists. The lowest intra-group agreement was seen between neonatologists. However, with the wide range in *κ* and *κ*_w_ values, there is only fair-to-moderate agreement for detecting signs of NEC according to DAAS in between groups of observers.

**Table 4:** Intra-group agreement

	*κ*			sd	*κ*-weighted			sd
	Mean	Min	Max		Mean	Min	Max	
G1^a^	0.2744	0.1032	0.7198	0.2315	0.3273	0.0198	0.8140	0.3025
G2^b^	0.2598	0.1373	0.3458	0.0874	0.4118	0.3126	0.5233	0.0791
G3^c^	0.3851	0.2179	0.6240	0.1501	0.5124	0.3278	0.8050	0.1644

^a^G1—radiology residents

^b^G2—neonatologists

^c^G3—pediatric radiologists

## Discussion

**Fig. 1:**
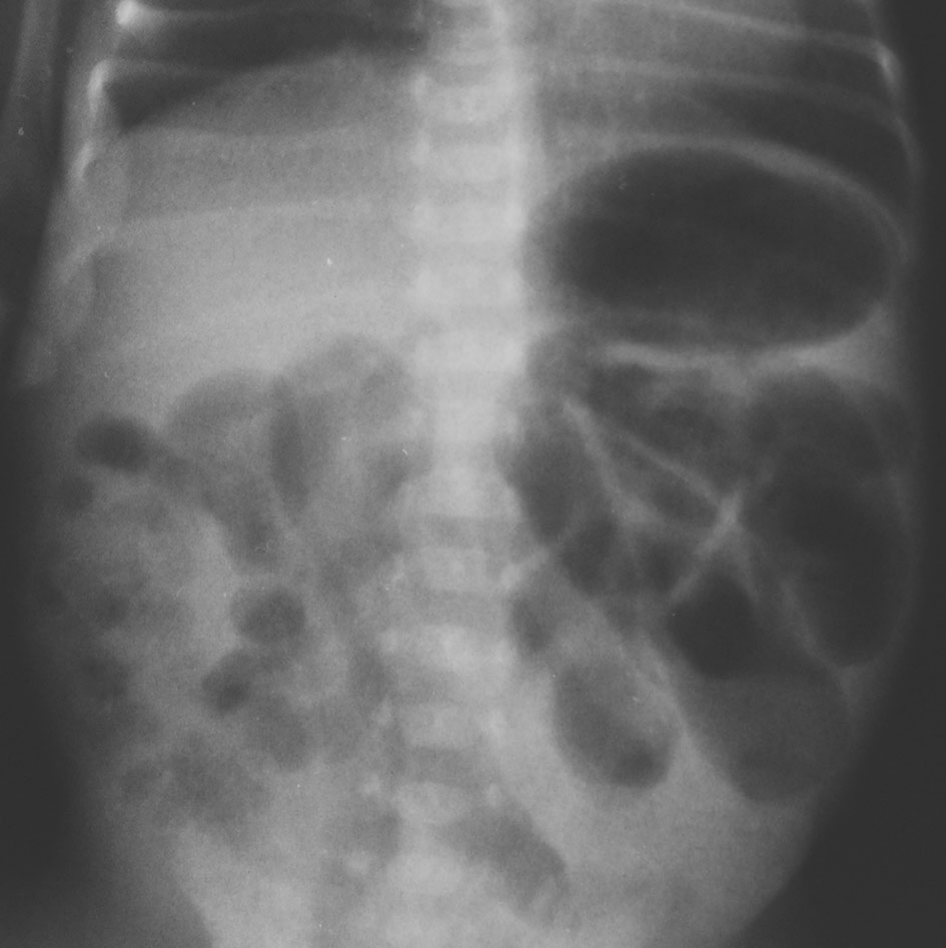
Abdominal radiograph shows diffuse gaseous intestinal distention with discrete signs suspected of pneumatosis in the lower right quadrant, six point according to DAAS scale—the example of highest variation between examiners (1, 2, 6, and 8)

**Fig. 2:**
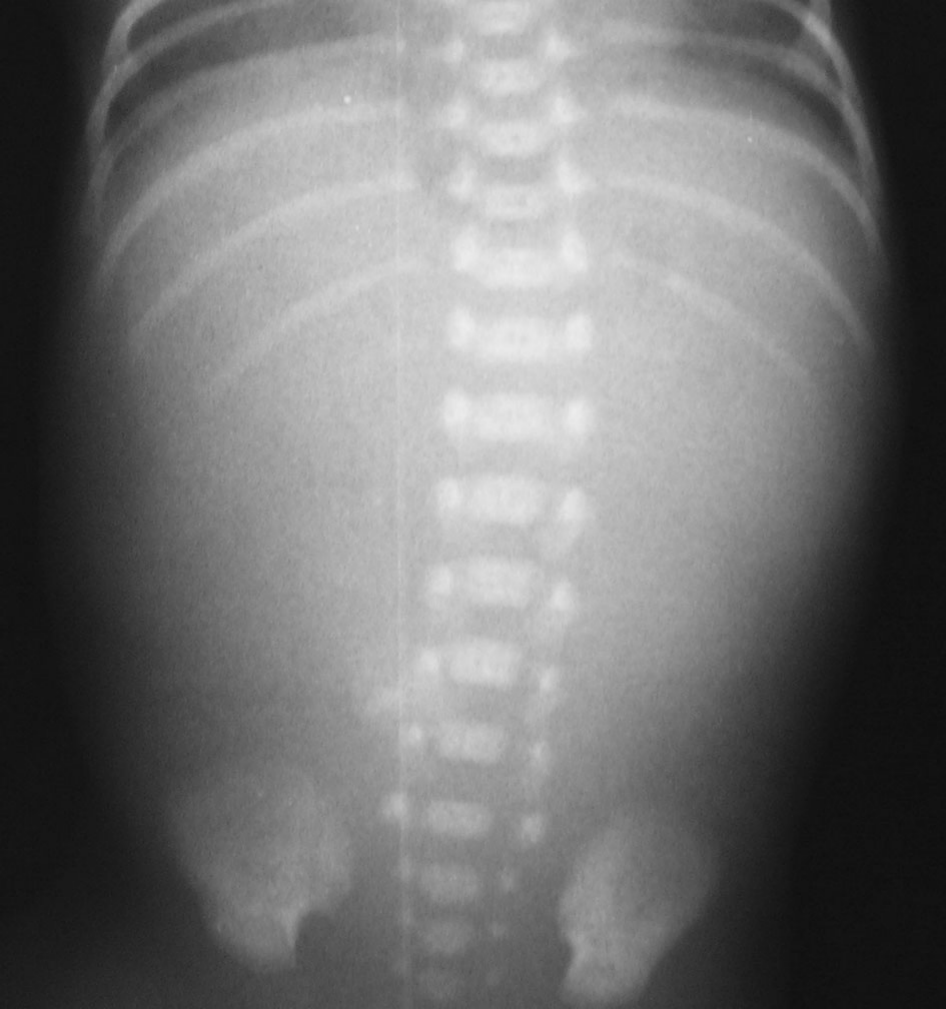
Radiograph demonstrates a gasless abdomen without findings of pneumoperitoneum; these findings cannot be classified according to DASS. However, according to the literature, it is the sign of occult perforation and advancing peritonitis

The early and correct diagnosis of necrotizing enterocolitis (NEC) is of utmost importance as this severe inflammatory process of the gastrointestinal tract in neonates and infants is still associated with high morbidity and mortality. Abdominal radiographs are the most widely accepted diagnostic imaging tool for the evaluation of neonates and infants with NEC or suspected NEC [17]. The radiological signs of occult perforation and advancing peritonitis are the presence of focal and diffuse gaseous intestinal distension (Fig. 1), air–fluid levels, bowel wall thickening, ascites, pneumatosis (Fig. 1), portal venous gas, pneumoperitoneum, and development of gasless abdomen (Fig. 2). According to the literature, bowel dilatation is present in 75–90% of cases of NEC, and focal or separated dilatation reflects mode-advanced disease [18]. In our group of neonates diagnosed as either definite or advanced NEC, the most common sign was pneumatosis and separated bowel dilatation.

Special attention should be paid to the sign referred to as a “persistent loop”, corresponding to affixed loop of bowel relatively unchanged in 24–36 h, as it may be a hallmark of impending perforation. The presence of portal venous gas is associated with severe disease and higher mortality rates with the specificity and positive predictive value of 100% for intestinal necrosis [18]. According to Tam et al. [19], the overall specificity and positive predictive values for the two predictors of perforation, pneumoperitoneum and development of gasless abdomen, are 92/88% and 92/82% in abdominal radiography, respectively. However, pneumoperitoneum, the only universally agreed sign that mandates surgical intervention, is present in only 50–75% of all neonates and infants with bowel perforation secondary to NEC [5, 20–22]. In our population, pneumoperitoneum was present in 2 (25%) out of 8 infants who underwent surgery. The main problem with radiological signs is that they might have a high positive predictive value (the highest values for pneumoperitoneum) but a very low sensitivity (less than 50%) [23].

The value of a diagnostic method is proved with the consistency of observation, which is also referred to standardization of reporting [24]. A recently introduced ten-point numeric scale of radiological signs in NEC—The Duke Abdominal Assessment Scale, is believed to be a solution in terms of standardization of the reporting terminology of radiographic findings. The scale also increases with disease severity and scores 7, 8, and 9 are highly associated with surgical intervention [13]. According to Coursey et al. [13], the previous studies available in literature, which did not use a standardization tool, such as DAAS, found poor inter-observer and intra-observer agreement in film interpretation. In their study, Coursey et al. [13] found substantial intra-observer and inter-observer agreement among study participants. The level of agreement was characterized by weighted *κ* values, which were reported by the authors at levels of 0.635–0.946 for the intra-observer agreement and 0.574–0.898 for the inter-observer agreement. Similarly, we obtained good to substantial levels of intra-observer agreement with *κ*_w_ values ranging from 0.4717 to 0.8140 with a single exception suggestive of poor reliability (*κ*_w_ 0.1830). However, with the wide range of *κ*_w_ values (0.0367–0.5353), we found only fair inter-observer agreement. Our results concerning inter-observer agreement were more comparable to those obtained by Rehan et al. [25]. Their study was conducted prior to the introduction of DAAS, and they examined the presence of intestinal distention, air–fluid levels, bowel wall thickening, portal venous gas, pneumoperitoneum, and the overall diagnosis of NEC. Rehan et al. characterized the level of inter-observer agreement with *κ* values and with a wide range of *κ* value they observed fair agreement for both radiological signs and the overall diagnosis of NEC (0.11–0.37 and 0.12–0.30, respectively) which is in concordance with our results at the levels from 0.0323–0.2920. Comparable results with the agreement for the radiographic diagnosis of suspected/confirmed NEC at the level of 0.31 (*κ* value) were presented by Napoli et al. [26]. Their study was conducted only amongst radiology practitioners.

The most recent study by El-Kady et al. [27] confirmed that there are differences in the inter-observer agreement between radiologists, pediatric surgeons, and trainees. The results of their study are similar to the ones obtained by us (*κ*_w_ values ranging from 0.51 to 0.87). They believe that it is reasonable to make efforts to improve compliance and adapt objective radiologic criteria, as well as to include alternative surveillance strategies for diagnosis of NEC [25].

As the study of Thuijls et al. [28] has shown, there are some promising new noninvasive markers for the early diagnosis of NEC. Finding a new noninvasive marker next to improvement of the evaluation system quality of abdominal radiographs could be a significant future perspective in the diagnostic evaluation and management of children with suspected NEC.

We recruited neonatologists as observers and, similar to the study of Rehan et al. [25], we found higher intra- and inter-+observer agreement in radiology practitioners, with the highest one among experienced pediatric radiologists. Despite the standardization of radiological signs of NEC, experience in the evaluation of abdominal radiographs in cases suspected of NEC is vital and the highest among pediatric radiologists. It is important to stress that the pediatric radiologists in the study performed only slightly less well than those in the Coursey et al. [13].

There are several limitations to our study that might have contributed to the poorer observer agreements. We selected a relatively small group of 87 radiographs for analysis. All were performed in anteroposterior projection in upright position, which resulted from an examination protocol of abdominal plain radiogram for the neonates in our institution, with consideration of the ALARA guidelines. Another limitation is that we included X-rays solely from one institution and that the study was conducted retrospectively with no immediate clinical impact of the diagnosis. We believe that many radiological features of NEC are subjective, and that the experience of the observers is a significant factor in the evaluation of the examinations. Therefore, the inclusion of non-radiology professionals as observers could have contributed to the observer variability. We did not compare the results of abdominal ultrasound studies, routinely performed in NICU patients, to X-ray reports in children with NEC or suspected NEC as it was not the aim of our study. However, in the view of recent studies by Muchantef et al. and Dilli et al. [8, 9], it is becoming clear that these two methods of imaging do complement each other, conveying data that may assist clinical decision making; although authors stress that further, prospective studies are necessary to fully assess the role of US in NEC.

In conclusion, we did not confirm that introduction of a numeric scale of radiological signs in the diagnosis of NEC or suspected NEC could facilitate reporting of abdominal radiographic findings. Besides, with high observer variability in interpretation of radiologic signs, DAAS appears to have limitations with respect to the radiological assessment of NEC.

## Electronic supplementary material

Below is the link to the electronic supplementary material.

Supplementary material 1 (XLS 33 kb)

Supplementary material 2 (XLS 30 kb)
